# The complete chloroplast genome sequence of *Thrixspermum amplexicaule* (Orchidaceae, Aeridinae)

**DOI:** 10.1080/23802359.2021.1959459

**Published:** 2021-09-24

**Authors:** Zu-Xia He, Ting-Zhang Li, Xi-Long Zheng, Yu-Feng Gu, Rui Zhang, Jian-Bing Chen

**Affiliations:** a Key Laboratory of National Forestry and Grassland Administration for Orchid Conservation and Utilization, Shenzhen, China; b The Orchid Conservation and Research Center of Shenzhen, Shenzhen, China; c School of Traditional Medicine Materials Resource, Guangdong Pharmaceutical University, Yunfu, China; d Shanghai Chenshan Plant Research Center, Chinese Academy of Sciences, Shanghai, China

**Keywords:** *Thrixspermum amplexicaule*, Orchidaceae, chloroplast genome, phylogenomics, phylogeny

## Abstract

The complete chloroplast genome sequence of *Thrixspermum amplexicaule* was assembled and analyzed in this work. The total chloroplast genome size of *T. amplexicaule* was 148,124 bp in length, containing a large single-copy (LSC) region of 86,079 bp, a small single-copy (SSC) region of 10,799 bp, and a pair of inverted repeat (IR) regions of 25,623 bp. The GC content of *T. amplexicaule* was 36.4%. It encoded a total of 120 unique genes, including 75 protein-coding genes, 37 tRNA genes, and eight rRNA genes. The results of phylogenetic analysis strongly supported that all four samples of *Thrixspermum* are monophyletic and *T. amplexicaule* was closely related to *T. centipeda*.

*Thrixspermum* Loureiro (1790), belonging to Orchidaceae, includes 161 species distributing in the tropical and subtropical regions from Asia to Australia, and only 16 species in China (Pridgeon et al 2014; Jin et al. 2019). *Thrixspermum* is close to *Phalaenopsis* based on the molecular phylogenetic study of Aeridinae (Zou et al. 2015). Up to now, only three complete chloroplast genomes of the genus were sequenced and published, including *T. centipeda, T. tsii* and *T. japonicum* (Ye et al. 2020; Chi et al. 2021). *Thrixspermum amplexicaule* (Blume) Rchb. f. (1867) is a special species that mainly distributing in China, India, Thailand, Vietnam, Malaysia, Indonesia, Philippines and Singapore (Lok et al. 2008; Jin et al. 2019), and Hainan Island of China is the northernmost boundary of the species in the world. Its flower color often varies from white to purplish and it takes only one day from blooming to falling which makes it difficult to meet pollinator in natural conditions (Jin et al. 2019). The species is rare and assessed as a near threatened (NT) species in China (Jin et al. 2019). Therefore, developing genomic resources for *T. amplexicaule* will provide basic information for further study on phylogeny, evolutionary biology and conservation biology.

In this study, the leaf sample of *T. amplexicaule* was collected from Zuntan Town, Haikou City, Hainan Province of China (110°17'11″E, 19°47'17″N, altitude 44 m). The specimens were deposited at the National Orchid Conservation Center (NOCC, Wen-Hui Rao: conservation@sinicaorchid.org) and Shanghai Chenshan Botanical Garden (CSH, Bin-Jie Ge: gebinjie123@163.com) under the voucher number Zheng XL et al. RAD137.

The genomic DNA was extracted from Silica gel dried leaf of *T. amplexicaule* and sequenced on the Illumina HiSeq platform by Shanghai Origin gene Biotechnology Company (Shanghai, China). DNA extraction, library constructing, sequencing and data filtering were reference in Liu et al. (2019). With the chloroplast genome of *T. centipeda* (MW057769) as a reference, the paired-end reads were filtered and assembled using GetOrganelle software (Jin et al. 2020). The assembled chloroplast genome was annotated with Geneious Prime (Biomatters Ltd., Auckland, New Zealand) (Kearse et al. 2012) and manual adjustment was conducted (Ye et al. 2020). The physical map of the chloroplast genome was generated using the online tool OGDRAW (http://ogdraw.mpimp-golm.mpg.de/) (Lohse et al. 2013). Finally, we obtained a complete chloroplast genome of *T. amplexicaule* and submitted to NCBI (Genbank accession MW574621).

The total chloroplast genome size of *T. amplexicaule* (MW574621) was 148,124 bp in length, containing a large single-copy (LSC) region of 86,079 bp, a small single-copy (SSC) region of 10,799 bp, and a pair of inverted repeats (IR) regions of 25,623 bp. The complete GC content of *T. amplexicaule* was 36.4%. It encodes a total of 120 unique genes, including 75 protein-coding genes, 37 tRNA genes, and eight rRNA genes.

To further understand the intergeneric and intrageneric phylogenetic relationship of *Thrixspermum*, the matrix of 12 species from Aeridinae and 3 outgroup species (*Calanthe lyroglossa*, *C. davidii* and *Changnienia amoena*) were aligned using MUSCLE version 3.8.31 software (Edgar 2004). The phylogenetic tree was constructed using the maximum likelihood method by IQ-TREE, and branch supports with the ultrafast bootstrap (Nguyen et al. 2015).

The phylogenetic analysis strongly support that *T. amplexicaule* was closely related to *T. centipeda,* and all four samples of *Thrixspermum* are monophyletic (BS = 100) (Figure 1). The results were consistent with morphological taxonomy and other phylogenetic study on Orchidaceae (Jin et al. 2019; Chi et al. 2021; Ye et al. 2020; Zou et al. 2015).

**Figure 1.**
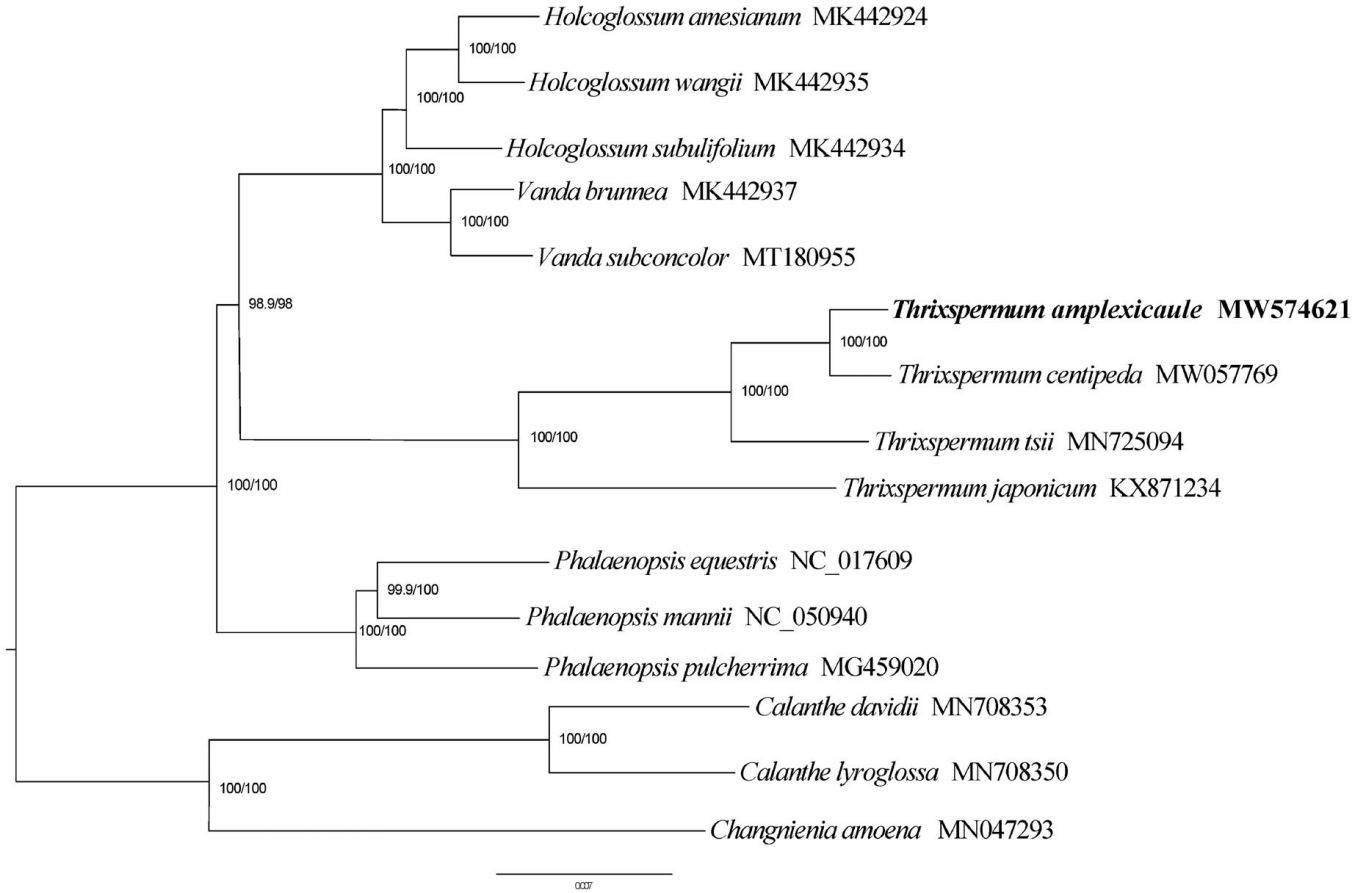
Maximum-likelihood phylogenetic tree based on 15 complete chloroplast genome sequences. Bootstrap support is indicated for each branch.

## Data Availability

The genome sequence data that support the findings of this study are openly available in GenBank of NCBI at [https://www.ncbi.nlm.nih.gov] (https://www.ncbi.nlm.nih.gov) under the accession no. MW574621. The associated ‘BioProject,’ ‘SRA,’ and ‘Bio-Sample’ numbers are PRJNA731595, SRR14618281, and SAMN19291068, respectively.
